# Commentary on Allaf et al.: Comparing countries with different legal cannabis markets can inform on the impact of regulating product type and potency

**DOI:** 10.1111/add.16312

**Published:** 2023-08-06

**Authors:** Martine Skumlien, Sam Craft

**Affiliations:** ^1^ Addiction and Mental Health Group (AIM), Department of Psychology University of Bath Bath UK

**Keywords:** cannabis, edibles, legalisation, poisoning, potency, THC

*Regulating the type and potency of cannabis products that can be sold in a legal market may influence the rate of cannabis‐related harms. Comparing countries with differing frameworks for legalisation can provide insight into effective strategies for minimising harm, and data must be routinely collected to monitor important health outcomes*.

The potential benefits and harms of legalising cannabis are hotly debated in many countries. However, these likely depend on the regulatory framework, such as whether restrictions are placed on product types and potency [1]. Comparing states that have taken different approaches to legalisation can help identify which strategies are effective for minimising cannabis‐related harms in a legal market.

In their systematic review, Allaf *et al*. [2] found that legalisation or decriminalisation of cannabis was followed by a rise in acute cannabis poisoning in paediatric patients, where it appeared to be driven in part by increased use and availability of edibles. Strict regulation on the manufacturing, sale and advertisement of edibles may, therefore, lower the risk of accidental exposure in the paediatric population. This could include mandating plain, child‐resistant packaging, prohibiting the sale of products that may appeal to children (e.g. gummies, animal‐shaped products) or that mimic existing trademarked confectionary products or banning the sale of edibles altogether [3].

Edibles and concentrates also typically have higher Δ^9^‐tetrahydrocannabinol (THC) potency compared with flower products [4]. Consuming higher potency cannabis is linked with increased risk of adverse effects, further amplifying the risk of poisoning with accidental exposure [5]. In a recent article, Hall *et al*. [6] propose that cannabis potency can be regulated in legal markets by (i) banning high‐THC products; (ii) capping THC content in cannabis products; or (iii) increasing cannabis taxes in proportion to THC content. Regulating products according to standard THC units (1 unit = 5 mg) should also be considered [7, 8]. However, greater restrictions on legal cannabis markets could also shift users into the illicit market if the latter can offer consumers greater product choice at lower prices, which would confer its own risks. Ultimately, research is still needed to establish how restrictions on potency, and the availability and prevalence of high‐potency cannabis products (including flower, edibles, concentrates and extracts), affect poisoning rates.

Research on the effectiveness of product restrictions for minimising cannabis‐related harm can benefit from the diverse regulatory approaches taken by different countries that have legalised cannabis. Only studies from the United States (US) and Canada, and one study from Thailand, were identified by the review by Allaf *et al*. [2]. However, many countries across Africa, Europe and South America, as well as the Australian Capital Territory, have decriminalised or legalised recreational or medicinal cannabis in the past decade. Myriad factors may influence the effect of legalisation or decriminalisation in any given country, such as the existing culture and typical practices around cannabis use and the degree to which public health factors are permitted to drive the regulatory framework.

For instance, Uruguay, the first country to fully regulate its recreational cannabis market, has taken a relatively restrictive and public health‐driven approach [9]. Uruguay allows sale of flower‐based products exclusively, which can only be obtained from a pharmacy, approved Cannabis Social Clubs, or home cultivation and with a yearly limit of 480 g per person. A cap of 9% THC applies to pharmacy cannabis, although the ban on extracts and edibles limits the de facto potency cap to the biological THC ceiling in cannabis flower, which is ~35% [10]. By contrast, the 23 US states that have legalised cannabis for recreational purposes to date have typically adopted more commercially driven models, allowing a wide range of product types and potency levels [11]. For instance, the sale of edibles is permitted in all 23 states, many of which have no legal restrictions on the permitted THC dosage per serving or package [12].

Uruguay and the US states of Colorado and Washington both legalised cannabis ~10 years ago, allowing for the comparison of longer‐term trends in two countries whose approaches to legalisation have differed substantially. Comparisons between these jurisdictions could serve as a useful guideline to other countries which are considering different frameworks for cannabis legalisation. However, it is noteworthy that in their review, Allaf *et al*. [2] did not identify any studies measuring changes in cannabis‐related poisonings following legalisation in Uruguay. It is essential that as more jurisdictions look to legalise cannabis through different regulatory frameworks, robust data collection systems are set up to monitor and evaluate the impact on important health indicators.

## AUTHOR CONTRIBUTIONS

**Martine Skumlien:** Conceptualization (lead); writing—original draft (lead); writing—review and editing (lead). **Sam Craft:** Conceptualization (supporting); writing—review and editing (supporting).

## ACKNOWLEDGEMENTS

None.

## FUNDING INFORMATION

MS is funded by a grant from the Engineering and Physical Sciences Research Council (EPSRC), grant number EP/V026917/1. SC is funded by grant MR/N0137941/1 for the GW4 BIOMED MRC DTP, awarded to the Universities of Bath, Bristol, Cardiff, and Exeter from the Medical Research Council (MRC).

## DECLARATION OF INTERESTS

The authors have no competing interests to declare.

## Data Availability

Data sharing not applicable to this article as no datasets were generated or analysed during the current study.
